# The expression and activity of Toll-like receptors in the preimplantation human embryo suggest a new role for innate immunity

**DOI:** 10.1093/humrep/deab188

**Published:** 2021-09-13

**Authors:** Wedad S Aboussahoud, Helen Smith, Adam Stevens, Ivan Wangsaputra, Helen R Hunter, Susan J Kimber, Mourad W Seif, Daniel R Brison

**Affiliations:** 1 Division of Developmental Biology and Medicine, Maternal and Fetal Health Research Centre, School of Medical Sciences, Faculty of Biology Medicine and Health, University of Manchester, Manchester Academic Health Sciences Centre, Manchester, UK; 2 Maternal and Fetal Health Research Centre, St. Mary’s Hospital, Manchester University NHS Foundation Trust, Manchester Academic Health Sciences Centre, Manchester, UK; 3 Division of Cell Matrix Biology and Regenerative Medicine, School of Biological Sciences, Faculty of Biology Medicine and Health, University of Manchester, Manchester Academic Health Sciences Centre, Manchester, UK; 4 Department of Reproductive Medicine, Old St. Mary's Hospital, Manchester University NHS Foundation Trust, Manchester Academic Health Sciences Centre, Manchester, UK

**Keywords:** innate immunity, ART, preimplantation, human embryo, cytokines, Toll-like receptor, infection

## Abstract

**STUDY QUESTION:**

Is the innate immunity system active in early human embryo development?

**SUMMARY ANSWER:**

The pattern recognition receptors and innate immunity Toll-like receptor (TLR) genes are widely expressed in preimplantation human embryos and the pathway appears to be active in response to TLR ligands.

**WHAT IS KNOWN ALREADY:**

Early human embryos are highly sensitive to their local environment, however relatively little is known about how embryos detect and respond to specific environmental cues. While the maternal immune response is known to be key to the establishment of pregnancy at implantation, the ability of human embryos to detect and signal the presence of pathogens is unknown.

**STUDY DESIGN, SIZE, DURATION:**

Expression of TLR family and related genes in human embryos was assessed by analysis of published transcriptome data (n = 40). Day 5 (D-5) human embryos (n = 25) were cultured in the presence of known TLR ligands and gene expression and cytokine production measured compared to controls.

**PARTICIPANTS/MATERIALS, SETTING, METHODS:**

Human embryos surplus to treatment requirements were donated with informed consent from several ART centres. Embryos were cultured to Day 6 (D-6) in the presence of the TLR3 and TLR5 ligands Poly (I: C) and flagellin, with gene expression measured by quantitative PCR and cytokine release into medium measured using cytometric bead arrays.

**MAIN RESULTS AND THE ROLE OF CHANCE:**

TLR and related genes, including downstream signalling molecules, were expressed variably at all human embryo developmental stages. Results showed the strongest expression in the blastocyst for *TLRs 9* and *5*, and throughout development for *TLRs 9, 5, 2, 6* and *7.* Stimulation of Day 5 blastocysts with TLR3 and TLR5 ligands Poly (I: C) and flagellin produced changes in mRNA expression levels of TLR genes, including the hyaluronan-mediated motility receptor (*HMMR*)*, TLR5, TLR7,* nuclear factor kappa-light-chain-enhancer of activated B cells (*NF-κB*) and monocyte chemoattractant Protein-1 (*MCP-1*) (*P* < 0.05, *P* < 0.001 compared to unstimulated controls), and release into culture medium of cytokines and chemokines, notably IL8 (*P* = 0.00005 and 0.01277 for flagellin and Poly (I: C), respectively).

**LIMITATIONS, REASONS FOR CAUTION:**

This was a descriptive and experimental study which suggests that the TLR system is active in human embryos and capable of function, but does not confirm any particular role. Although we identified embryonic transcripts for a range of TLR genes, the expression patterns were not always consistent across published studies and expression levels of some genes were low, leaving open the possibility that these were expressed from the maternal rather than embryonic genome.

**WIDER IMPLICATIONS OF THE FINDINGS:**

This is the first report of the expression and activity of a number of components of the innate immunity TLR system in human embryos. Understanding the role of TLRs during preimplantation human development may be important to reveal immunological mechanisms and potential clinical markers of embryo quality and pregnancy initiation during natural conception and in ART.

**STUDY FUNDING/COMPETING INTEREST(S):**

This work was funded by the Ministry of Higher Education, The State of Libya, the UK Medical Research Council, and the NIHR Local Comprehensive Research Network and NIHR Manchester Clinical Research Facility and the European Union’s Horizon 2020 Research and Innovation Programmes under the Marie Skłodowska-Curie Grant Agreement No. 812660 (DohART-NET). In accordance with H2020 rules, no new human embryos were sacrificed for research activities performed from the EU funding, which concerned only in silico analyses of recorded time-lapse and transcriptomics datasets. None of the authors has any conflict of interest to declare.

**TRIAL REGISTRATION NUMBER:**

n/a.

## Introduction

Fundamental functions of the body’s immune system are recognition of microbes and responses to those that are pathogenic (Beutler, 2009). The Toll-like receptor (TLR) family has been identified as the main family of pathogen recognition receptors (PRRs) (Medzhitov and Janeway, 1997; Medzhitov *et al*., 1997; Lemaitre *et al*., 2012) and mediates the innate immune response, which is the first line of host defence (Beutler, 2009). PRRs include many families, such as the nucleotide-binding oligomerization domain (NOD)-like receptors (NLRs) and the retinoic acid-inducible gene-1, RIG-1-like receptors (RLRs). Following pathogen detection, these receptors trigger tissue- and pathogen-specific biological responses (Medzhitov *et al*., 1997; Koval'chuk *et al*., 2011; Mukherjee and Lukacs, 2013). There are 22 human NLR genes (Ariffin and Sweet, 2013) and a substantial body of evidence points to NLRs as key regulators of early mammalian embryogenesis and reproduction, in particular NLRP-2, 5 and 7 (Van Gorp *et al*., 2014).

TLRs are widely expressed throughout the female reproductive tract (Beutler, 2009) and may mediate interactions between the reproductive and immunological systems in events such ovulation and pre-eclampsia (Riley and Nelson, 2010). The epithelial lining of the human endometrium expresses TLRs 1–10 and is the first layer to provide defence against pathogenic invasion involving approximately 30 different types of sexually transmitted infections (Wira *et al*., 2015). Even in the absence of infection during normal pregnancy, the uterine epithelium has to permit and accept the implanted and growing semi allogenic embryo (Wira *et al*., 2005). This requires specific modulation of endometrial epithelial immunity, such that for successful implantation to take place, three essential items are required; receptive endometrium, competent embryo and immune system modulations mediating the reciprocal interactions between them.

TLRs respond to a variety of pathogenic signals, including single- and double-stranded viral RNA, flagellin from both Gram-positive and Gram-negative flagellated bacteria, and lipopolysaccharide (LPS) from gram negative bacteria (Uematsu and Akira, 2008), to produce immunomodulatory responses. TLR3 responds to double- stranded viral RNA (dsRNA) (Alexopoulou *et al*., 2001) to produce type one interferon (IFN), which has immunostimulatory effects, and also to a synthetic ligand, the poly inosinic-poly cytidylic acid (poly (I: C)), commonly used in research investigating TLR3 activity. TLR5 responds to flagellin stimulation to activate proinflammatory responses (Hayashi *et al*., 2001). The signalling pathway can be dependant or independent of the key adaptor molecule myeloid differentiation factor (MyD88); all TLRs except TLR3 can utilise this molecule (Akira and Takeda, 2004).

Maternal LPS infection during early pregnancy in mice has been shown to suppress embryonic implantation, probably as a result of a TLR-mediated inflammatory immune response from the endometrium (Deb *et al*., 2004b, 2005; Jaiswal *et al*., 2006). Maternal LPS exposure also alters preimplantation embryonic growth and cell lineage allocation prior to implantation, with adverse effects on health of subsequent offspring including behaviour, adiposity and an altered innate immune response (Williams *et al*., 2011). However, it is not known whether the maternal infection acts solely at the level of an endometrial TLR response, and there has been little consideration of whether early embryos themselves might possess a functional system for recognising and signalling the presence of pathogens in the female tract. A recent study investigated TLR expression in human oocytes and granulosa cells from primordial and primary ovarian follicles and showed that TLR gene expression was positive for TLRs 3, 4 and 5: the authors concluded that human primordial and primary follicles express genes that would provide them with the ability to interact with innate immune proteins during follicle activation (Ernst *et al*., 2020).

Preimplantation embryos are known to be highly sensitive to surrounding environmental conditions while undergoing critical early developmental events (Monk *et al*., 2019): these include embryonic genome activation, epigenome remodelling, and the first cell differentiation, in which the blastocyst differentiates into inner cell mass (ICM), which will give rise to the embryo, and trophectoderm (TE), which forms placenta (Dobson *et al*., 2004). Within the context of human ART, embryos are exposed to an artificial *in vitro* environment and despite precautions taken during manufacturing and in the clinic, the presence of impurities in embryo culture media cannot be excluded (Morbeck *et al*., 2014), including cell proteins and microbial structures and toxins. Even in very small amounts, these can stimulate the innate immune receptors and initiate an immune response (Verthelyi and Wang, 2010) or detrimental signalling pathways (Wang *et al*., 2004; Kragstrup *et al*., 2015; Wittmann *et al*., 2015). Therefore, this study was aimed at exploring whether the preimplantation human embryo possesses functional elements of the innate immunity system, which might have a physiological role in immunomodulation during implantation and could enable the embryo to detect and signal the presence of pathogens. We show that human embryos express TLRs and other families of innate immunity genes in oocyte, 4-cell, 8-cell (intact and individual blastomeres), blastocyst, ICM and TE samples. The data support proof of principle that TLRs 3 and 5 in Day 5 human blastocysts are functional by stimulating them with their specific ligands poly (I: C) and flagellin, respectively, and showing changes in cytokine mRNA expression levels and gene expression profile.

## Material and methods

### Human embryos

Embryos unsuitable or surplus for treatment from current (fresh) or previous (frozen) IVF cycles were obtained with informed written consent from patients at Old St. Marys Hospital, Manchester or other IVF units in the north-west of England, in accordance with ethics approval from the National Research Ethics Service committee south central (Berkshire) (Research Ethics Committee reference: 12/SC/0649), and a research license from the Human Fertilisation and Embryology Authority (HFEA; R0026), centre 0067 (Old St. Mary’s Hospital; fresh embryo research) and University of Manchester (0175; frozen-thawed embryo research). Embryos frozen at either the pronuclear or early cleavage stages were thawed using ThawKit Cleave (Vitrolife, Gothenburg, Sweden) according to the manufacturer’s instructions. Embryos were cultured in G1 and G2 sequential media or GTL continuous culture medium (Vitrolife, Gothenburg, Sweden) to D-6 post-fertilisation and graded using a standardised scheme (Cutting *et al*., 2008; Embryology, 2011). Embryos were only used if they scored ≥3 for blastomere size and degree of fragmentation, and their speed of development was normal. Photomicrographs were taken using a Leica light microscope (Leica DM IL LED, Leica, Leicester, UK) and blastocysts lysed on the 5th (D-5) or 6th (D-6) day following fertilisation.

### Expression of TLR and related molecules during preimplantation development

We used published microarray data to investigate the expression of TLRs 1–10 and related signalling molecules in human oocyte, 4-cell stage embryos, 8-cell stage embryos and blastocysts, comparing our own in house data (Shaw *et al*., 2013) to three other published microarray data sets (Zhang *et al*., 2009; Xie *et al*., 2010; Vassena *et al*., 2011). Expression was estimated from the hgu133plus2.0 (1–3) and the hugene1.0 ST exon tiling Affymetrix array platforms from individual embryos (Vassena *et al*., 2011; Shaw *et al*., 2013), and from pooled embryos (Zhang *et al*., 2009; Xie *et al*., 2010). The array quality metrics package in R was run as described in Smith *et al*. (2019) to exclude arrays with unacceptable levels of noise or technical faults. At least three replicate samples were analysed at each stage. For each embryo microarray, probe set expression was ranked and turned into a centile of total expressed probe sets in comparison to negative and positive control genes. In cases where multiple probe sets match a given gene, expression of that gene was averaged across the probe sets. This approach (Assou *et al*., 2011) allows standardised comparisons between arrays. Positive (Ubiquitin C (*UBC*)*,* ß-Actin (*ACB*)*,* Zona Pellucida 1 (*ZP1*)), and negative (Immunoglobulin J (*IGJ*)) control genes were included for comparison. Frozen Robust Microarray Analysis (fRMA) was applied for normalization of the studies, as in Smith *et al*. (2019). As a result, we were able to look at trends in expression that were consistent across studies, array platforms, replicates and embryo developmental stages.

We next examined expression of TLR and related molecules in a more detailed microarray developmental series consisting of: oocytes, 4-cell embryos, 8-cell embryos, individual 8-cell stage blastomeres, blastocysts, and separated TE and ICM samples (n = 4 samples for oocyte, 4-cell, 8-cell and blastocyst, with one of the 8-cell embryos disaggregated into eight individual blastomeres, and six paired ICM and TE samples) isolated from blastocysts (Smith *et al*., 2019). Microarray data were normalised with *Mas5* (Smith *et al*., 2019), allowing comparison between different groups. The lowest level for considering positive gene expression was set at a threshold of 5.64; values <5.64 were considered as no expression, values >5.64 positive gene expression (Ruane *et al*., 2020). We validated our microarray expression data by using gene-specific quantitative PCR (Q-PCR) to confirm mRNA expression of *TLR*s 3, 5, 6, 7 and 9 and *TRAF6, MCP-1, NFKBIA* and *NLRP-1*, in three additional D-5 human blastocysts (data not shown).

### Stimulation of human D-5 blastocysts with TLR3 and TLR5 ligands

To study the activity of TLR3 and TLR5 in D-5 human embryos, their specific ligands poly (I: C) (InvivoGen, San Diego, CA, USA) and flagellin (FLA-ST Ultrapure, from *Salmonellatyphimurium*, InvivoGen, San Diego, CA, USA), were added to embryo culture media. Fifteen human embryos at pronucleate (PN) or early cleavage (2–4-cell; EC) stage were thawed on two different occasions and cultured in G1 media (Vitrolife, Gothenburg, Sweden) to D-3 and G2 media (Vitrolife, Gothenburg, Sweden) to D-5. D-5 blastocysts were then treated with 0.5 or 1 μg/ml poly (I: C), 50 or 100 ng/ml flagellin, or G2 medium alone (control) for 24 h. Poly (I: C) and flagellin concentrations used were taken from the literature (Le Tortorec *et al*., 2008; Aboussahoud *et al*., 2010), as well as the manufacturers’ instructions, taking into consideration that poly (I: C) is a potent TLR3 activator. D-6 blastocysts were lysed for PolyAPCR (see below), while the 24 h supernatants were collected, centrifuged at 10,000*g* for 5 min at 4°C, transferred to fresh tubes and stored at −80°C.

### Cytokine bead array assay

To characterise any elevation in cytokines and chemokines in response to flagellin or to Poly (I: C), cell-free supernatants were analysed using a multiplex Cytometric Bead Array (BD Biosciences, San Jose, CA, USA). Beads internally dyed with varying intensities of a proprietary fluorophore and coated with capture antibodies specific to a cytokine or chemokine were incubated with 25 µl of supernatant for 1 h. A secondary phycoerythrin labelled antibody (25 µl) was then added and incubated for 2 h. The beads were then washed, and samples were analysed by a FACSArrayTM Bioanalyser (San Jose, CA, USA). The data were analysed with FCAP ArrayTM software provided by Soft Flow, Inc., Burnsville, MN, USA, and sample concentrations were determined for IL-1α, IL-1β, IL-6, IL-8, IL-10, monocyte chemoattractant Protein-1 (MCP-1) and tumour necrosis factor (TNF). The manufacturer states that the working assays range for most analytes is 10–2500 pg/ml, and the analysis laboratory has demonstrated that for the current experiment analytes, the level of sensitivity is 0–2500 pg/ml (Sue Clark, Flow Cytometry Core Facility, Medical School, University of Sheffield, personal communication). For the second experiment a more sensitive assay was used where the level of sensitivity for IL-8 and IFN-g was 0–2500 fg/ml.

### Blastocyst lysis, reverse transcription and global amplification (PolyAPCR)

Individual D-6 blastocysts were lysed and reverse transcribed as described previously (Brady and Iscove, 1993; Bloor *et al*., 2002). Briefly, each embryo was removed from its culture drop and put in a UV irradiated tube containing 10ul of lysis mix. Subsequently, the tube was heated to 65°C for 1 min followed by 25°C for 3 min followed by reverse transcriptase M-MLV (GibcoBRL) for first strand synthesis. PolyAPCR was performed to amplify mRNA as described by Brady and Iscove (Brady and Iscove, 1993; Brady, 2000). The expression of β-actin in blastocysts was considered as the minimum inclusion criterion following our standard protocols (Kimber *et al*., 2008). Normalisation of the double-stranded polyA cDNA samples was performed using the PicoGreen assay according to the manufacturer’s instructions (Invitrogen, Carlsbad, CA, USA) to perform the Q-PCR with the same concentration of 1 ng/µl used for all samples.

Quantitative real-time PCR was performed using the prepared cDNA from blastocysts. Primers were designed using Primer-BLAST (Ye *et al*., 2012) to amplify target genes within 500 bps immediately following the polyadenylation signal (Supplementary Table SI). All samples were run in triplicate. Results were analysed using CFX Manager Version 3.1 (Biorad, Hercules, CA, USA). Three blastocysts were used for each gene detection analysis. All analysis of data was pooled from three independent biological experiments, with three replicates per sample.

### Statistical analysis

Microarray data were imported into GraphPad Prism version 6.0 Software (Hearne Scientific Software, Victoria, Australia), normalised with Mas5 and analysed using the Kruskal–Wallis one-way ANOVA by rank test. For Q-PCR data, the expression level of genes were generally moderate to low (CT 24-34) and varied between blastocysts, therefore were analysed using the comparative CT method, as previously used in our group (Bloor *et al*., 2002). Briefly, the difference in cycle time (ΔCT) was determined as the difference between the number of cycles required for amplification of the test gene and the reference housekeeping gene, human ß-actin. For signals which were quantified relative to βactin mRNA, the 2^−^^ΔΔCt^ method was used (Livak and Schmittgen, 2001). The mean ΔCt values for each sample triplicate was calculated and imported into GraphPad Prism version 6.0 Software (Hearne Scientific Software, Victoria, Australia). The results were expressed as mean ± SEM. Statistical analysis was performed by using ANOVA with Tukey’s multiple comparison test. *P* < 0.05 was considered significant.

## Results

### Expression of TLR family and downstream signalling genes in human preimplantation embryos

As an initial screen for the expression of *TLR* and other *PRR* family member genes and signalling pathways, we analysed our global gene expression microarray dataset from a developmental series of individual oocytes, 4- and 8-cell and blastocyst stage embryos (Shaw *et al*., 2013). Comparing percentiles of expression in comparison to positive control genes *UBC, βactin* (both expressed though development) and *ZP1* (oocyte specific), and a negative control gene *IGJ*, allowed assessment of relative expression levels of *TLR* genes (Table I).

**Table I:** Expression of Toll-like receptor genes in human preimplantation embryos.

Gene*	Probe set IDs	Oocyte	4-cell	8-cell	Blastocyst
*UBC*	208980_s_at	99.45	99.51	99.39	99.59
*ACTB*	AFFX-HSAC07/X00351_3_at, AFFX-HSAC07/X00351_5_at, AFFX-HSAC07/X00351_M_at	82.31	87.58	84.17	89.63
*ZP1*	237335_at	93.71	88.28	77.34	71.95
*TLR9*	223903_at	68.14	69.48	86.06	57.73
*TLR5*	210166_at	66.67	77.29	39.18	73.95
*TLR2*	204924_at	50.11	68.9	40.82	31.35
*TLR6*	207446_at, 239021_at	39.9	56.31	44.39	42.68
*TLR7*	220146_at, 222952_s_at	48.85	48.81	31.45	46.69
*TLR8*	220832_at, 229560_at	30.75	21.25	28.02	49.27
*TLR10*	223750_s_at	25.7	40.49	37.09	20.02
*TLR4*	1552798_a_at, 221060_s_at, 232068_s_at	24.18	25.15	40.05	20.54
*TLR1*	210176_at	14.14	16.51	25.2	9.14
*IGJ*	212592_at	2.17	3.53	5.52	2.53

* Toll-like receptors (TLRs) 1–10 together with positive (Ubiquitin C; (UBC), Actin Beta; (ACTB), Zona Pellucida Glycoprotein 1; (ZP1)) and negative (immunoglobulin J polypeptide; IGJ) control genes: data from Shaw *et al.* (2013). ≤20th percentile = white= no expression; >20th and <40th percentile= light grey= weak expression; ≥40th and <70th percentile = dark grey= moderate expression; ≥70th percentile = black= strong expression. For technical reasons related to the arrays it was not possible to include TLR3 in this comparison.

The array data showed high expression for *TLR 9*, moderate for *TLRs 5, 2, 6* and *7* with low expression for *TLRs 8, 10* and *4* and no expression for *TLR1*. A search of published data was carried out and comparison made with those on embryos of similar developmental stage (Xie *et al*. 2010; Zhang *et al*., 2009; Vassena *et al*., 2011). This confirmed that, in comparison to the same negative and positive control genes, *TLRs 5* and *9* are among the highest expressed TLR genes during early human embryo development, robust to microarray platform and source of embryos. Our Shaw *et al.* (2013) data also showed expression of TLR signalling genes, including Interferon Regulatory Factor 3 (*IRF3*)*,* TIR domain-containing adapter molecule 1(*TICAM1*)*,* Nuclear factor-kappa-B inhibitor alpha (*NFKBIA*)*,* and NF-kappa-B inhibitor epsilon (*NFKBIE*)*;* moderate expression for Interleukin-1 receptor-associated kinase 4 (IRAK4), Tumour necrosis factor receptor (*TNFR*)*-associated factor 6* (*TRAF6*)*,* TIR Domain Containing Adaptor Protein (*TIRAP*)*, T*ranslocation Associated Membrane Protein* 1*(*TRAM1*)*,* Nuclear Factor Kappa B Subunit 1(*NFKB1*) and, Nuclear Factor Kappa B Subunit 2(*NFKB2*); and minimal expression for T cell differentiation protein (*MAL*)*,* Myeloid differentiation factor 88 (*MYD88*)*,* and*,* TANK Binding Kinase 1 (*TBK1*) (Table II).

**Table II:** Expression of TLR signalling molecules in human preimplantation embryos.

Gene	Probe set IDs	Oocyte	4-cell	8-cell	Blastocyst
*MAL*	204777_s_at	45.66	42.46	29.5	39.34
*MYD88*	209124_at	21.8	34.2	43.48	25.29
*IRAK4*	219618_at	50.66	73.77	53.81	69.7
*IRF3*	202621_at	86.96	95.18	87.76	90.13
*TRAF6*	205558_at	71.04	60.7	84	57.77
*TBK1*	218520_at	23.02	35.96	31.52	20.45
*TICAM1*	213191_at	82.6	86.56	78.35	83.41
*TIRAP*	1552804_a_at, 1554091_a_at	45.67	50.13	52.74	50.01
*TRAM1*	201398_s_at, 201399_s_at	35.34	39.86	44.34	63.33
***NFKB1***	209239_at	59.1	83.5	23.86	68.49
***NFKB2***	207535_s_at, 209636_at	67.87	72.04	74.34	49.42
***NFKBIA***	201502_s_at	97.7	83.86	89.81	83.91
***NFKBIE***	203927_at	78.02	95.84	74.09	89.48

* TLR signalling genes, data from Shaw *et al*. (2013). T cell differentiation protein **(***MAL*), Myeloid differentiation factor 88 (*MYD88*), Interleukin-1 receptor-associated Kinase 4 (*IRAK4*), Interferon Regulatory Factor 3 (*IRF3*), Tumour necrosis factor receptor (TNFR)-associated factor 6 (*TRAF6*), TANK Binding Kinase 1 (*TBK1*), TIR domain-containing adapter molecule 1 (*TICAM1*), TIR Domain Containing Adaptor Protein (*TIRAP*), Translocation Associated Membrane Protein 1 (*TRAM1*), Nuclear Factor Kappa B Subunit 1 *(NFKB1*), Nuclear Factor Kappa B Subunit 2 (*NFKB2*), Nuclear factor-kappa-B inhibitor alpha (*NFKBIA*), NF-kappa-B inhibitor epsilon (*NFKBIE*); ≤ 20th percentile = white= no expression; >20th and < 40th percentile= light grey= weak expression; ≥ 40th and <70th percentile = dark grey= moderate expression; ≥ 70th percentile = black= strong expression.

**Figure 1:**
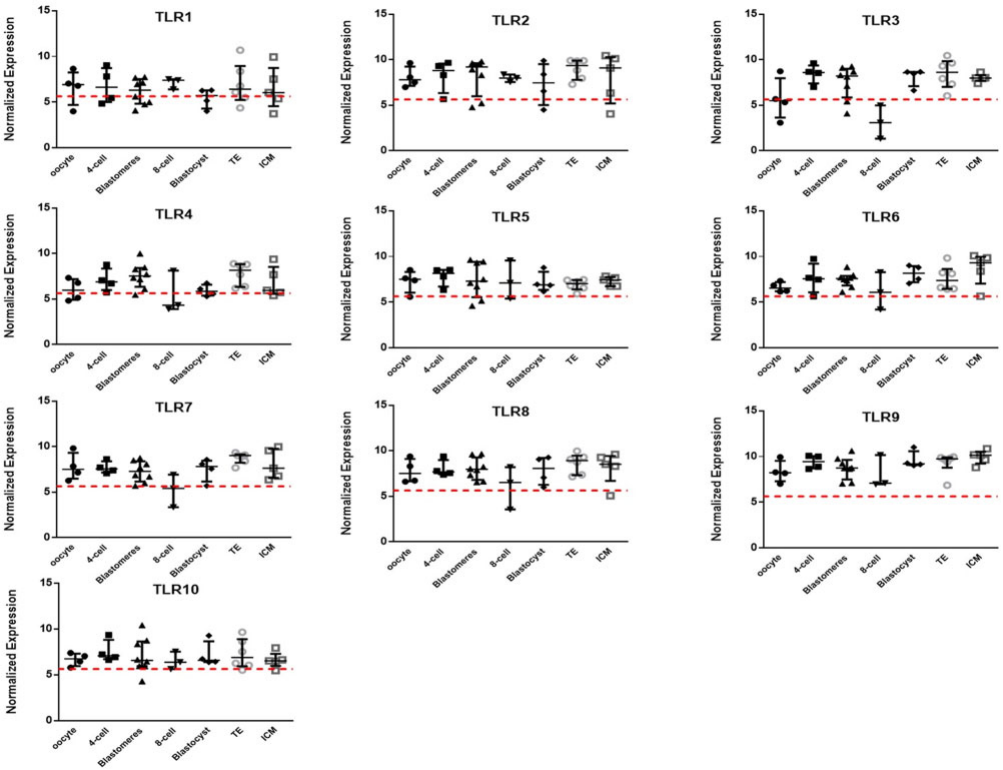
**Expression of *TLRs 1*–*10* in human embryos**. Microarray data (Smith *et al*., 2019) showing the relative expression of Toll-like receptors (*TLRs*) 1–10 in individual human preimplantation embryos from oocyte to blastocyst (n = 3 at 8 cell, n = 4 for all other stages), in isolated 8-cell stage blastomeres (blastomeres n = 8) and in trophectoderm (TE) and inner cell mass (ICM) samples isolated from blastocysts (n = 6 paired samples). Microarray data were normalised with *MAS 5* and the threshold level for gene expression above background was set at 5.64 (dashed horizontal line); values <5.64 are considered as no expression, values >5.64 are positive gene expression. Data are presented as the mean ± SEM.

The expression of the main *TLR* genes in a second in-house embryo developmental series of individual oocytes, 4-cells, 8-cell blastomeres, intact 8-cell embryos, blastocysts, and separated TE and ICM samples from Smith *et al*. (2019) is shown in Fig. 1. Expression levels of *TLRs 2, 5* and *9* were again higher in comparison to relatively low expression of *TLRs 1, 4* and *10* (compare to Table I). There were no significant changes in expression during development except for *TLR3*, which was expressed significantly more strongly in blastocysts (and isolated ICM and TE), and 4 cell embryos, compared to oocytes and 8-cell embryos (Fig. 1, *P* < 0.05). Mechanical separation of an 8 cell embryo into blastomeres and blastocysts into ICM and TE samples did not seem to affect *TLR* expression in comparison to the intact embryos (Fig. 1). We also looked at the equivalent previously published human embryo single cell RNAseq datasets (Yan *et al*., 2013; Petropoulos *et al*., 2016). Yan *et al*. (2013) showed expression of *TLR3* at moderate levels up to the 8-cell stage, with *TLR5* expressed throughout development. The data from Petropoulos *et al*. (2016), re-analysed by our group (Smith *et al*., 2019), is of particular interest for expression in various cell lineages at the blastocyst stage, and shows expression of *TLRs5*,*7* and *8* in various subpopulations of cells (Supplementary Fig. S1).

**Figure 2:**
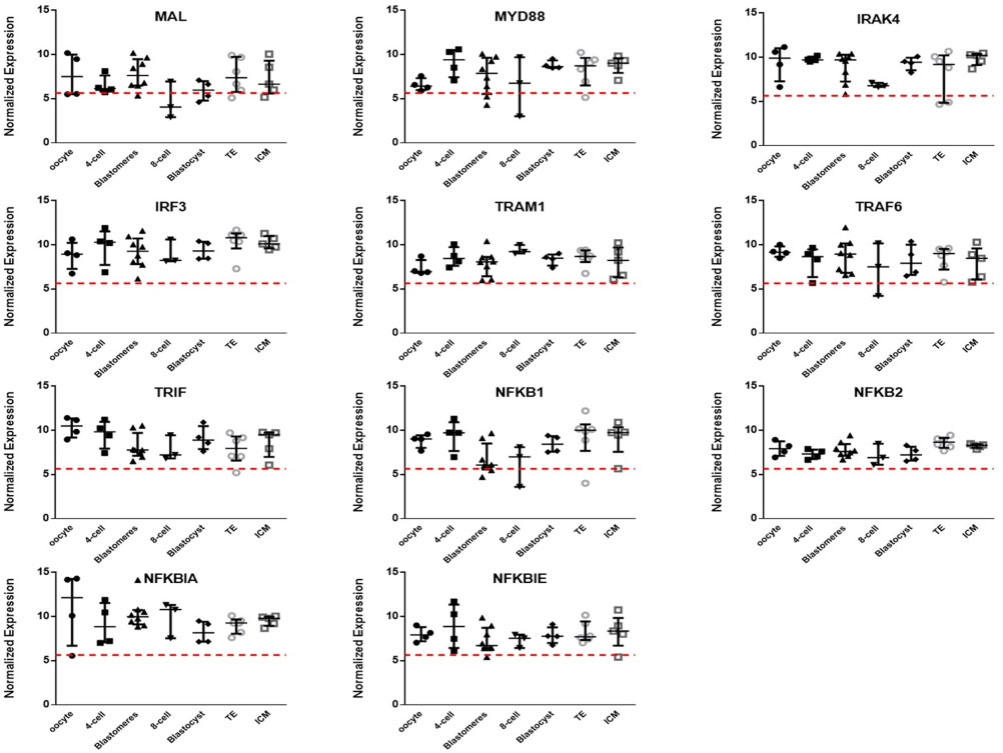
**Expression of TLR signalling molecules in human preimplantation embryos.** Microarray data (Smith *et al*., 2019) showing the relative expression of the main TLR signalling molecules in individual human preimplantation embryos from oocyte to blastocyst (n = 3 at 8 cell, n = 4 for all other stages), in isolated 8-cell stage blastomeres (blastomeres, n = 8) and in TE and ICM samples isolated from blastocysts (n = 6 paired samples). Microarray data were normalised with MAS 5 and the threshold level for gene expression above background was set at 5.64 (dashed horizontal line); values <5.64 are considered as no expression, values >5.64 are positive gene expression. Data are presented as the mean ± SEM.

The main TLR signalling molecules: *MAL, MyD88, IRAK4, IRF3, TRAM1, TRAF6, TRIF, NF-κB, NFKBIA* and *NFKBIE* were also expressed in this series (Fig. 2). *NFKBIA, IRAK4, TRAF6, TRIF, NFKB1, NFKB2* and *NFKBIE* showed a high level of expression, moderate expression was detected with *MAL, TRAM1*, and the lowest level of expression with *MyD88*. Again, 8-cell isolated blastomeres and ICM and TE showed similar expression to intact 8-cell embryos and blastocysts, respectively (Fig. 2 and Supplementary Fig. S1).

We also analysed the expression of NLR and RLR families, and found that *NOD1, NOD2, NOD3, NOD4, NOD5, NLRP1, NLRP2, NLRP3* and *IPAF* genes were expressed throughout preimplantation development (Supplementary Fig. S2).

There was also positive gene expression of *RLR1, RLR2* and *RLR3* (Supplementary Fig. S2). The *NLRP2* gene showed the highest expression level amongst the TLR, NLR and RLR genes. Finally, we showed that human embryos expressed a range of cytokines, which are downstream of TLR signalling, including IL-8 (Supplementary Fig. S3).

### Responses to poly (I: C) and flagellin treatment measured by cytometric bead array: towards developing a TLR function assay for human embryos

**Figure 3:**
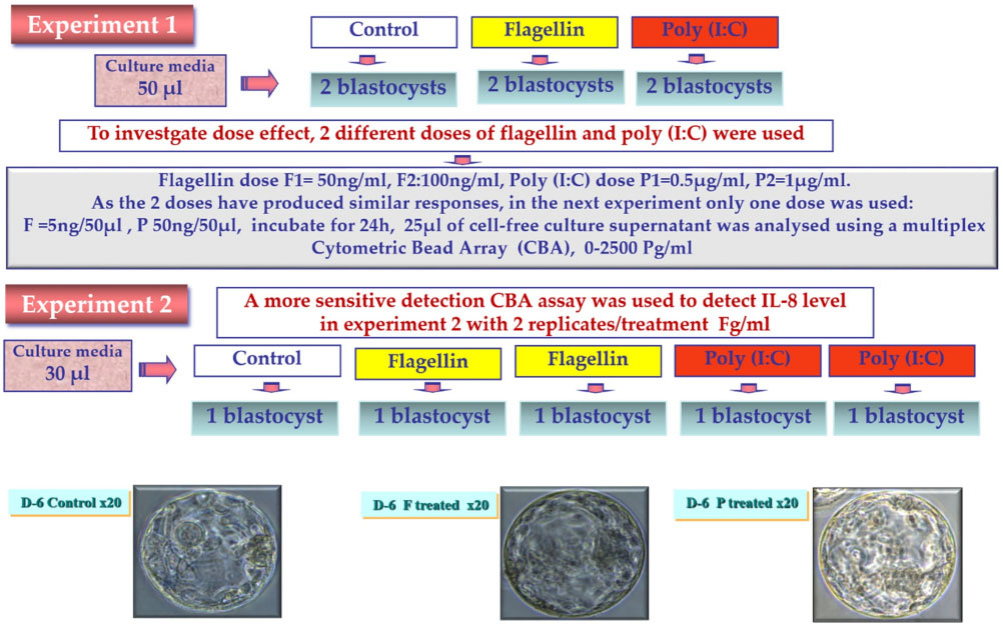
**Two-stage experiment to develop a cytokine/chemokine response assay for single human blastocysts using a cytometric bead array.** Experiment 1: Paired Day 5 (D-5) blastocysts exposed to two different concentrations of flagellin or poly(I: C) and supernatants analysed by cytometric bead array (CBA) array. Experiment 2: Single D-5 blastocysts exposed to flagellin or poly(I: C) and supernatants analysed for IL-8 production. Images show bright-field microscopy of representative D-6 blastocysts prior to lysis for gene expression analysis.

Having established human embryo mRNA expression for a range of TLRs, related signalling molecules and downstream cytokines, we went on to ask whether this system might be active, by culturing embryos in the presence of known stimulants of TLR5 (flagellin) or TLR3 (poly (I: C) (Fig. 3).

**Figure 4:**
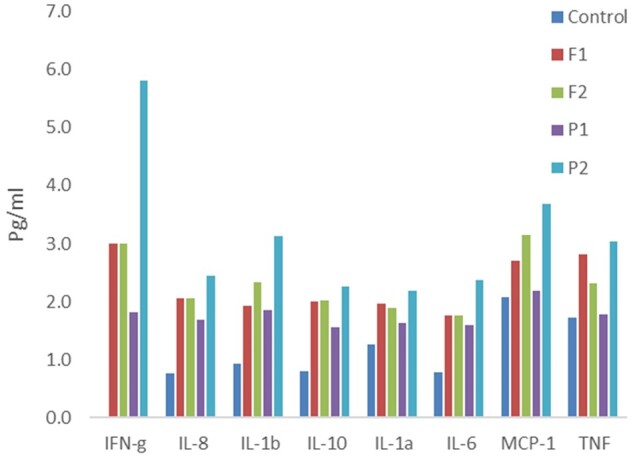
**The effect of flagellin or poly (I: C) treatment on cytokine/chemokine production measured by CBA.** Control = no treatment (2 blastocysts). F1 = Flagellin 50 ng/ml, 2 blastocysts; F2 = Flagellin 100 ng/ml, 2 blastocysts; P1= Poly (IC) 0.5 μg/ml, 2 blastocysts, P2= Poly (IC) 1 μg/ml, 2 blastocysts. CBA, cytometric bead array.

To develop a cytokine response assay for single human embryos, a total of 16 D-5 blastocysts were allocated to two experimental groups, with no significant difference in blastocyst morphological grade or degree of expansion between the control, flagellin and poly (I: C) treated blastocysts (Fig. 3). In experiment 1, under control conditions, D-5 human embryos produced all of the cytokines measured (IL-6, -8, -10, -1B, -1a, TNF and MCP-1) except INF-g, in detectable amounts (1–5 pg/ml) into culture medium. The flagellin and Poly (I: C)-challenged embryos produced elevated levels of each cytokine, particularly at the higher poly (I: C) concentration (Fig. 4). The most striking increase was that of INF-g, which was not seen in control media but was detected at high levels in both concentrations of flagellin and Poly(I: C). IL-8, IL-1b, IL-10 and IL-6 all showed at least 2-fold higher levels in response to both flagellin and Poly(I: C) (Fig. 4).

**Figure 5:**
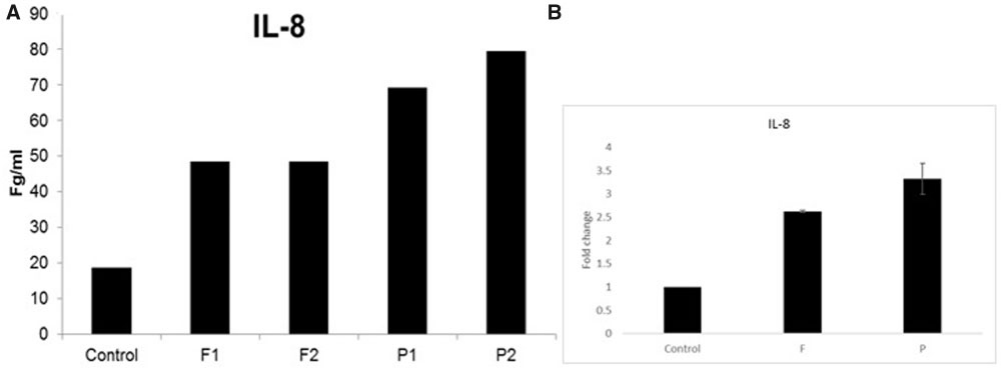
**The effect of flagellin or poly (I: C) treatment on IL-8 production by single blastocysts.** Control = no treatment. F1 = F2 = Flagellin 100 ng/ml, P1 = P2 = Poly (IC) 1 μg/ml,. (**A**) Il-8 production. (**B**) Fold change in IL-8 concentration in media of blastocysts exposed to Flagellin (F) and Poly (IC) from Experiments 1 and 2 (Figs 4 and 5A) combined (n = 4 drops containing n = 6 blastocysts analysed per condition; data are mean ± SEM relative to control levels set at 1.00; *P* = 0.00005 and 0.0128 for F and P, respectively).

To confirm these findings, in Experiment 2, we analysed single blastocysts in 30 ul drops of control media, flagellin (100 ng/ml), or Poly (I: C) (1ug/ml), with a more sensitive CBA assay for IL-8 in the fg range (Fig. 5). This showed a 2–3-fold increase in IL-8 production in both flagellin and Poly (I: C) (Fig. 5A). Combined for analysis, these experiments show that IL-8 production occurred in each of four replicates in response to both flagellin and Poly (I: C) at 2.5- and 3.3-folds, respectively (*P* = 0.00005 and 0.01277, respectively) compared to controls (Fig. 5B).

### Gene expression responses in human D-6 blastocysts in response to flagellin and poly (I: C) treatment

**Figure 6:**
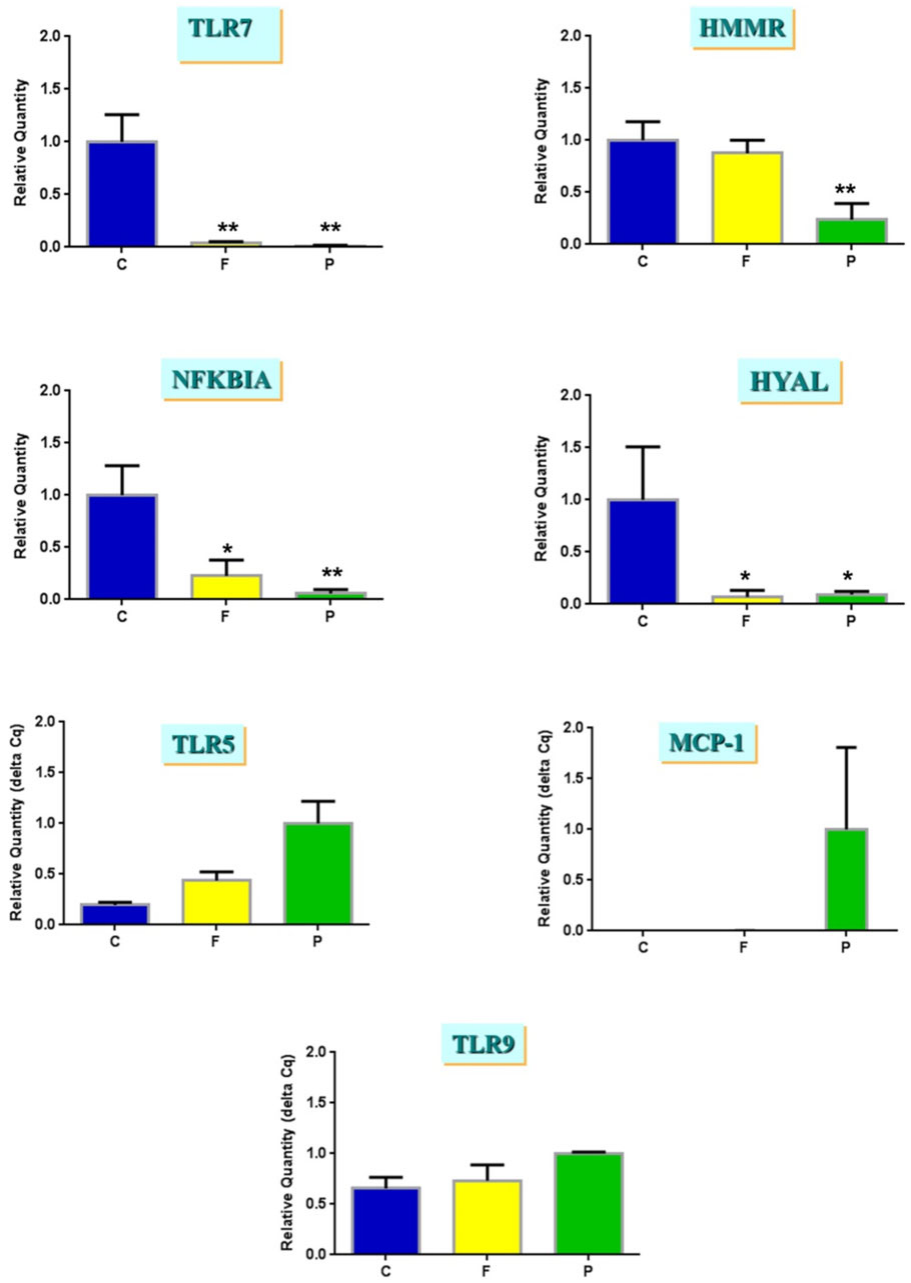
**D-6 blastocyst gene expression in response to flagellin (F) or poly (I: C) (P) treatment.** Data are presented as mean ± SEM of relative quantitative expression values in three D-6 blastocysts on each of three occasions. *P < 0.05, **P < 0.001 compared to untreated controls.

As the cytokine assays were operating near the limit of sensitivity, we confirmed the response of human blastocysts to flagellin and poly (I: C) treatment by analysing expression of a number of key TLR target genes in the exposed and control blastocysts using PolyAPCR (Fig. 6). The expression of *TLR7, NFKBIA* and *HYAL* was significantly (*P < 0.05, **P < 0.001) reduced in the presence of flagellin (100 ng/ml), and Poly (I: C) (1 µg/ml), compared to control embryos. The expression of *HMMR* was reduced, while that of *TLR5* and *MCP-1* was increased, in poly (I: C) only, while *TLR9* expression was unchanged in either condition (Fig. 6).

## Discussion

**Figure 7:**
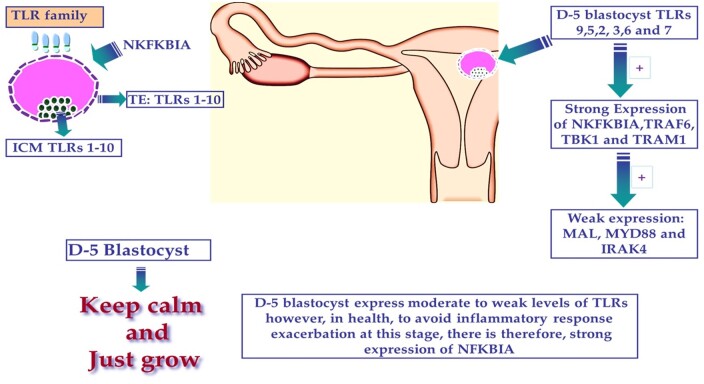
**Expression and possible role of TLRs in human pre-implantation development**.

To our knowledge, this is the first study to demonstrate the expression and activity of the TLR system in human preimplantation embryos. We show embryonic gene expression for many of the main components of the TLR system, including a range of receptors, key signalling molecules and inflammatory cytokines, from the oocyte stage onwards and in most cells and compartments of the developing embryo including the ICM (which goes on to form the foetus) and the TE (which gives rise to the placenta). Furthermore, our data show that TLR system is active and potentially functional, with human blastocysts showing clear changes in gene expression and secreting inflammatory cytokines into the culture medium in response to TLR-ligands (Fig. 7).

Our data, and other published studies, showed strongest expression in the blastocyst for *TLR9* and *5*, and throughout development for *TLRs 9, 5, 2, 6* and *7.* Expression of TLRs showed wide variation among embryos, and some variability between published human embryo datasets, which could be attributed to variation in genetic background as well as to environmental factors such as embryo culture; such heterogeneity in expression in human embryos is well established (Shaw *et al*., 2013; Smith *et al*., 2019). Complex interactions between genetic variation and the environment may impact the susceptibility of the organism to invading pathogens (Arbour *et al*., 2000; Schroder and Schumann, 2005; Netea *et al*., 2012). For example, in dog endometrium the expression of *TLR4* was higher in the presence of a uterine bacterial infection (Chotimanukul and Sirivaidyapong, 2011). Despite the variability noted, human embryos show a distinct pattern of TLR expression. The high level of expression of *TLR9* might reflect a need to respond to the presence of invading pathogens and other environmental factors; our finding that *TLR9* mRNA expression is high under control conditions and not upregulated by flagellin or poly(I: C) is consistent with a system in ‘on’ mode. *TLR4*, which is generally considered one of the most important TLR family members, was expressed only weakly in our development series and not in other embryo datasets we analysed. Tissues such as ectocervix and vagina also lack *TLR4* expression, and the authors postulated that this might be a protective mechanism against undesired excessive inflammatory response to the commensals in the vagina (Fazeli *et al*., 2005). *TLR3* was expressed up to the 8-cell stage in the Yan *et al*. (2013) study, but more strongly in 4-cells and blastocysts in our data (Smith *et al*., 2019). We hypothesised that *TLR* expression might be higher in the blastocyst TE cells which mediate the interaction with the external environment, including contact with pathogens and with endometrial cells during implantation; however, we noticed no significant difference in *TLR* expression in TE compared to ICM. Little data exist regarding the expression and possible function of *TLRs* in preimplantation embryos. However, a recent study performed on zebrafish and mouse embryos demonstrated the existence of an innate immune function during the earliest stages of embryonic development (Hoijman *et al*., 2021). It seems that human embryos in early developmental stages might be exposed to pathogen invasion, therefore are able to call upon innate immunity molecules. Similarly, the epithelial cells lining the fallopian tube are the first line of defence against pathogenic exposure that might invade the tube: for example, infection with *Chlamydia trachomatis* is considered as the leading cause of tubal complications that might cause infertility and tubal ectopic pregnancy (Hafner, 2015). Noticeably, *TLRs* modify the tubal response to chlamydial infection (Al-Kuhlani *et al*., 2020). Mouse cumulus oocyte complexes express *TLRs 2, 4, 8* and *9* (Shimada *et al*., 2006, 2008) and abnormal *TLRs* expression in human cumulus cells has an impact on embryo quality in patients with polycystic ovary syndrome (Gu *et al*., 2016). Human oocytes also show expression of *TLR* proteins (Ernst *et al*., 2020), which may be transcribed from maternal stored mRNA, including possibly that transferred across subzonal bridges from the cumulus cells. Another study showed that during maturation of human oocytes, there is a marked alteration in composition of the proteome and secretome which is directed for homeostasis, cellular attachment, and environment interaction (Virant-Klun *et al*., 2016). Human sperm have also been shown to express *TLRs 2* and *4*, which are functional (Fujita *et al*., 2011). However, there are no similar investigations of human embryos. Human embryonic stem cells (hESCs) derived from the blastocyst ICM show strong expression of *TLR5*, as well as *TLRs 1, 3, 4* and *6,* with *TLRs 7* and *9* at low levels, and *TLR 8* and *10* undetectable (Foldes *et al*., 2010). This is substantially different to the expression pattern we see in native ICM, however in common with ICM, hESC lines also showed a functional *TLR5* response to flagellin (Foldes *et al*., 2010). Human embryos expressed a range of the other PRRs, including *NLR* and RLR families, however the level of expression was generally low for all receptors other than *TLRs*. *NLRP2* was the highest expressed receptor among *NLR, RLR* and *TLR* families and it is noteworthy that the cluster of *NLRs* that contains *NLRP2, 4, 5, 7, 8, 9, 11, 13* and *14* members has been associated with roles in reproduction and early stages of mouse embryonic development (Van Gorp *et al*., 2014). Levels of mRNA expression of *NLRs* that relate to reproduction and early stages of embryonic development fell sharply between fertilization and the blastocyst stage, in common with other oocyte-derived maternal effect genes (Li *et al*., 2010, 2013).

Human embryos also express the main *TLR* signalling molecule In humans, the Nuclear factor kappa-light-chain-enhancer of activated B cells

(*NF-κB*) family is central to transduction of extra-cellular signals from many receptors, such as *TLR* family, into many cellular activities; physiological and pathological (Honey *et al*., 2005). Several *NF-κB* isoforms and inhibitors of *NF-κB* (*IκB*) have been identified, which mainly block the nuclear localization and transcriptional activity of *NF-κB* (Verma *et al*., 1995; Jacobs and Harrison, 1998; Basak *et al*., 2007). In our data, one of these isoforms, *IκBα* (*NKKBIA*), is the highest expressed signalling gene across all of the embryo development series examined. *MAL, MYD88* and *IRAK4* showed lower levels of expression in our data, but are known to be activated in response to *TLR* stimulation and the key adaptor molecule *MyD88* showed some upregulation in 8-cell and blastocyst embryos. In contrast, most of the other signalling molecules showed a similar level of expression across development (*IRAK4, MAL, TRAF6, TRAM, NF-κB* and *IRF3*).

*TLR* signalling can also alter the expression of pro-inflammatory cytokines and chemokines that activate and attract, respectively, immune cells to contribute to normal physiological homeostasis in the human endometrium (Kayisli *et al*., 2002; Fahey *et al*., 2005). Infection at early stages of pregnancy can dramatically alter the level of cytokines and growth factors involved in the process of implantation and embryo development, and may be a cause of early implantation failure and pregnancy loss (Deb *et al*., 2004a, 2005; Jaiswal *et al*., 2006; Robertson *et al*., 2018). Our study shows that human embryos express inflammatory cytokines and chemokines, some of which, including *IL-8* and *MCP-1*, are elevated in D-5 blastocysts compared to earlier stages, whereas some inflammatory molecules, such as *IL-1α*, *IL-1β* and *CCR3*, showed no change with development. *IL-18*, which plays a role during embryo implantation (Ledee *et al*., 2006), was significantly up-regulated in blastocysts in comparison to oocytes.

Having provided evidence that the key components of the *TLR* system are expressed in human embryos, we considered if this system might be active and potentially functional, by testing the response of human embryos to bacterial ligands for *TLRs* 3 and 5. We show using a sensitive CBA bead array that a range of cyto/chemokines are increased in the culture medium of human blastocysts treated with flagellin and Poly(I: C). *IFN-g* is heavily upregulated, being produced only by stimulated blastocysts, not in control medium, and the IL family overall also shows consistent upregulation. Since the CBA array was working at the limits of sensitivity with such small numbers of cells, we repeated the experiment with a more sensitive Fg range assay for *IL-8*. This confirmed the *IL-8* response, with overall a highly consistent increase seen, providing proof of principle of a cyto/chemokine response by human embryos to flagellin and Poly(I: C). We note, however, that caution should be taken as the cytokines produced after treatment with flagellin and poly (I: C) strongly suggest, but do not prove, that the effect is specific. Lastly, we show significant changes in the expression of a number of genes in D-6 blastocysts following exposure to flagellin and Poly(I: C). Four of the seven genes in our screen (*NFKBIA, TLR7, HMMR* and *HYAL*) showed reduced expression levels, and interestingly, all but one were reduced by both ligands, with *HMMR* being supressed only by poly (I: C). *TLR9* expression was similar in control, flagellin and poly(I: C) groups, and a stimulatory effect was seen only with poly(I: C), with *TLR5* and *MCP-1* showing clearly increased levels. The downregulation of the inhibitor *NFKBIA* in response to *TLR3* or *TLR5* stimulation in other tissues allows for inflammatory defence responses to take place by activating the cytoplasmic NF-f (Dolcet *et al*., 2005; Paciolla *et al*., 2011), and together with the increases in *TLR5* and *MCP-1*, is consistent with the normal host response to elevate its innate immunity defence mechanism upon exposure to microbes (Carpentier *et al*., 2005). The reduction in expression of one of the main hyaluronic acid (HA) receptors (*HMMR/RHAMM*) and the hyaluronidase gene (*HYAL1*) responsible for degrading HA into low MW trophic signally forms, suggests a general reduction in embryonic responsiveness to the presence of HA. This is of interest since HA plays vital roles during the embryo implantation process and is added to embryo culture medium in human ART (Fouladi-Nashta *et al*., 2017; Ruane *et al*., 2020). Thus, our data are consistent with a reduction in HA-mediated embryo implantation potential in the presence of a bacterial or viral infection. In general, the overall effect of flagellin and poly (I: C) on gene expression is suppressive, which is difficult to interpret in terms of host defence mechanisms; especially the persistent very low expression of *TLR7* which in other systems is upregulated to increase the detection capacity of the host innate immunity against invading pathogens. The only stimulatory effect was seen with poly (I: C), which signals via *TLR3*, and not with flagellin/*TLR5*. The level of IL-8 produced by blastocysts is also somewhat higher in response to poly (I: C) in comparison to flagellin. *TLR3* is an intracellular receptor and responds to dsRNA that is commonly a product of viral replication inside body cells, for which poly (I: C) is a synthetic mimic. In the current experiment, poly (I: C) was applied to the embryo culture media, and this application has created changes at the gene level as well as at the cyto/chemokine level. This indicates that D-5 blastocysts have the ability to transport the poly (I: C) from outside the blastocyst across the apical surface of the TE, although the mechanism is unknown. Generally speaking, activation of intracellular *TLRs* 3, 7 and 8 by their cognate ligands is achieved by phagocytosis, pinocytosis or via receptor-mediated endocytosis (Chaturvedi and Pierce, 2009). However, other possibilities cannot be excluded during this early stage of human embryo development.

Our data suggest that the innate immunity system is expressed and active in the human preimplantation embryo (Fig. 7). The expression of *TLRs* in human embryos is not entirely clear, since published studies are not consistent and expression of some *TLRs* is low, leaving open the possibility of inheritance from the Maternal l rather than the embryonic genome. Regardless of this, known ligands for *TLR3* and *TLR5*, widely used to mimic bacterial and viral infections, respectively, elicit clear changes in gene expression and the production of cyto/chemokines by individual human embryos.

## Conclusion

Our data suggest that pathogens have the capacity to alter embryo quality and developmental competence directly, and perhaps also to signal an inflammatory response to the maternal tract and so modulate the implantation process and the initiation of pregnancy. Further investigations will be required to determine whether the *TLR* stimulation response is effective during the embryonic journey in the fallopian tube, when any embryonic signals released might be diluted, and/or in the uterus during endometrial attachment.

A pro-inflammatory environment has been shown to be conducive to successful implantation, however, our data on embryos suggest a balance between suppression and stimulation of the innate immunity response. This may reflect the need for embryo survival in the presence of pathogens, versus the need for the maternal tract to respond to infection. Irrespective, human embryos at the time of implantation appear to have the potential to respond directly to and contribute to the maternal inflammatory immune response following exposure to a pathogenic invasion. This has implications for both natural conception and outcomes of ART, since infections can occur in the maternal tract and in ART media used for embryo culture and transfer to the uterus. There has been much focus on the role of ART media in implantation success and the long-term health of children (Chronopoulou and Harper, 2015; Sunde *et al*., 2016). Our data now suggest that an innate immune response to pathogens should form part of this consideration and should also prompt calls to explore embryo culture media components for their immunogenicity prior to their clinical use. Embryonic responses should now be taken into consideration in parallel with mucosal maternal innate immunity. In particular, understanding the role of the *TLR* family during human preimplantation development may be important to reveal immunological mechanisms and potential clinical markers of embryo quality and pregnancy initiation during natural conception and in ART.

## Data availability

The microarray data used in this article have all been published previously and are available in the public respositories listed in each of the publications cited. The individual embryo RT-PCR data underlying this article cannot be released in full owing to confidentiality issues surrounding the use of human embryos in research, however, a summary of the data will be made available on reasonable request to the corresponding author.

## Supplementary Material

deab188_Supplementary_Figure_S1

deab188_Supplementary_Figure_S2

deab188_Supplementary_Figure_S3

deab188_Supplementary_Table_S1
