# Supplementary material for: The expression and activity of Toll-like receptors in the preimplantation human embryo suggest a new role for innate immunity
Source: Hum Reprod. 2021 Sep 13;36(10):2661–75. doi: 10.1093/humrep/deab188 (PMC8450873; doi:10.1093/humrep/deab188)
Supplement: deab188_Supplementary_Figure_S1 [file deab188_supplementary_figure_s1.pdf]

Human Reproduction, pp. 1–1, 2021
doi:10.1093/humrep/deab188

human reproduction

SUPPLEMENTARY DATA

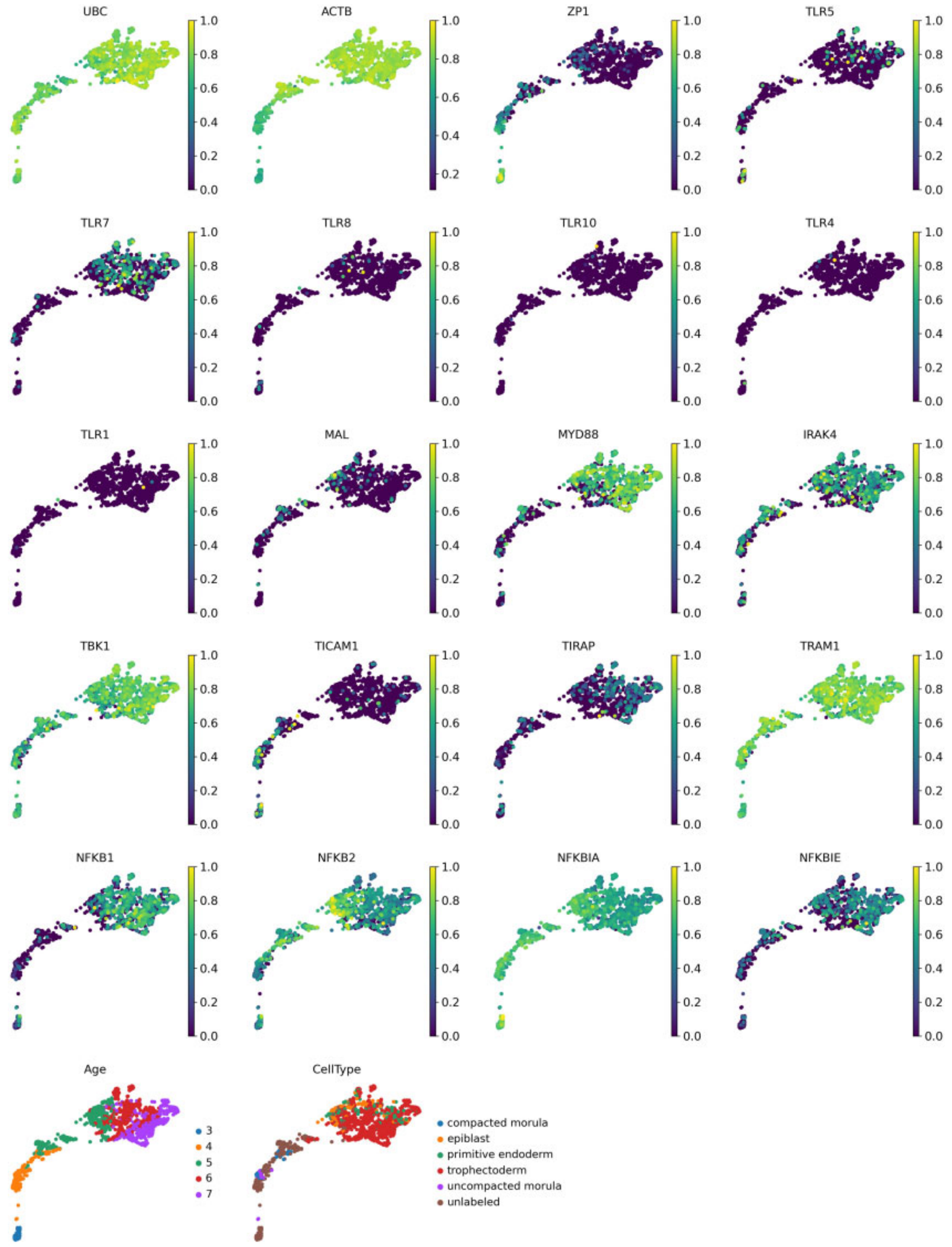

Supplementary Figure S1 Expression levels of TLRs and related genes in individual cells of human morulae and blastocyst stage embryos. Data are from Petropoulos et al. (2016). Raw reads were downloaded from ArrayExpress (E-MTAB-3929) and trimmed using Trim Galore! (https://www.bioinformatics.babraham.ac.uk/projects/trim_galore/) using the default parameters for reads from Illumina platforms. The reads were then aligned against the hg38 reference genome using HISAT2 (Kim et al., 2019) and the resulting features were quantified using Velocyto.py (La Manno et al., 2018). CPM normalised counts of the features were calculated and the visualisation above generated using Scanpy (Wolf et al., 2018).

Human Reproduction, pp. 1–1, 2021
doi:10.1093/humrep/deab188

SUPPLEMENTARY DATA

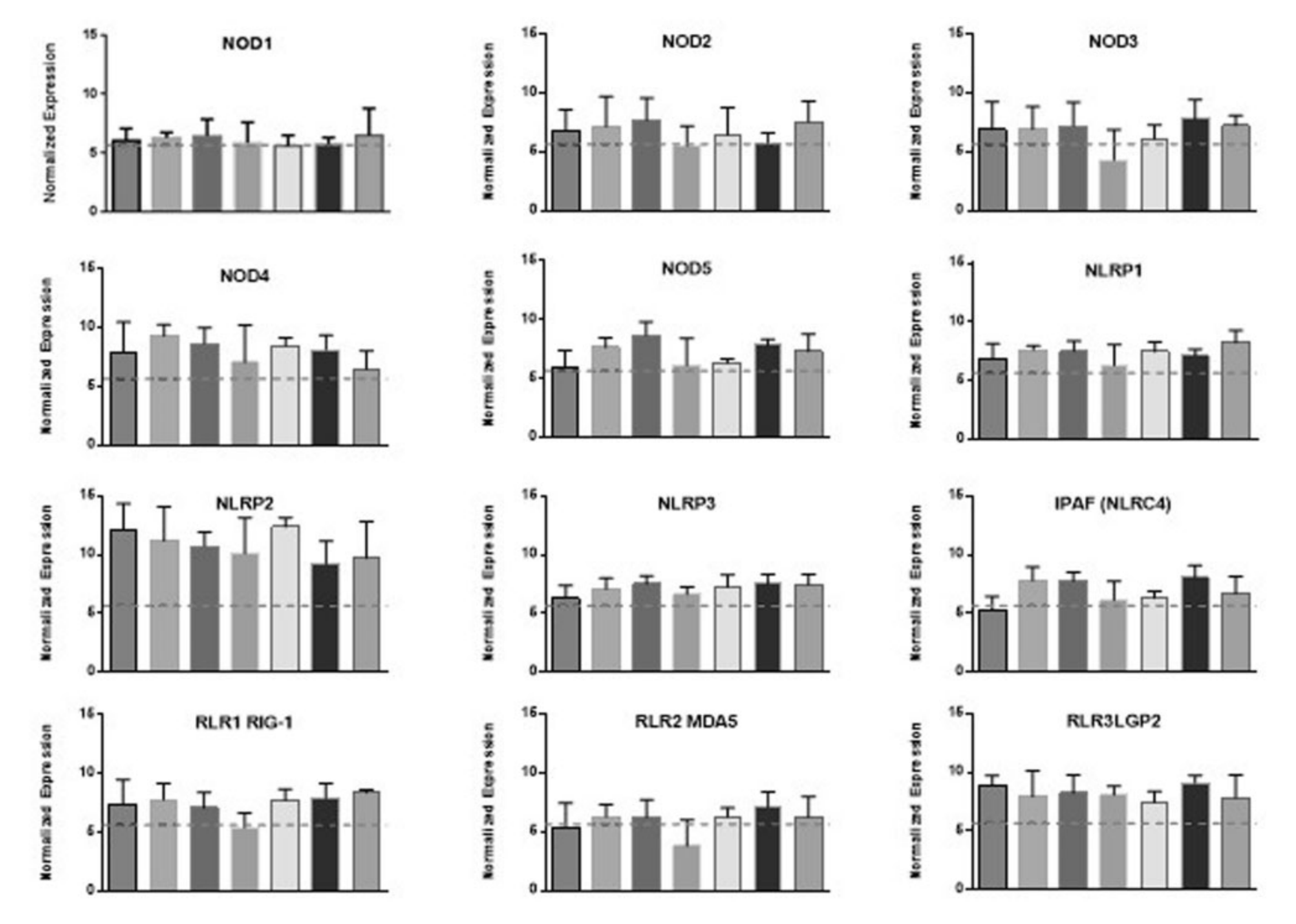

Supplementary Figure S2 Expression of NLR and RLR genes in human embryos. Microarray data (Smith et al., 2019) showing the relative expression of the NLR (nucleotide-binding oligomerization domain (NOD)-like receptor) and RLR (retinoic acid-inducible gene-1, RIG-1-like receptor) molecules in individual preimplantation human embryos from oocyte to blastocyst (n=3 at 8 cell, n=4 for all other stages), in isolated 8 cell stage blastomeres (blastomeres n=8) and in trophectoderm (TE) and inner cell mass (ICM) samples isolated from blastocysts (n=6 paired samples). Microarray data were normalised with MAS 5 and the threshold level for gene expression above background was set at 5.64 (dashed horizontal line); values <5.64 are considered as no expression, values >5.64 are positive gene expression. Data are presented as the mean ± SEM.

Human Reproduction, pp. 1–1, 2021
doi:10.1093/humrep/deab188

human reproduction

SUPPLEMENTARY DATA

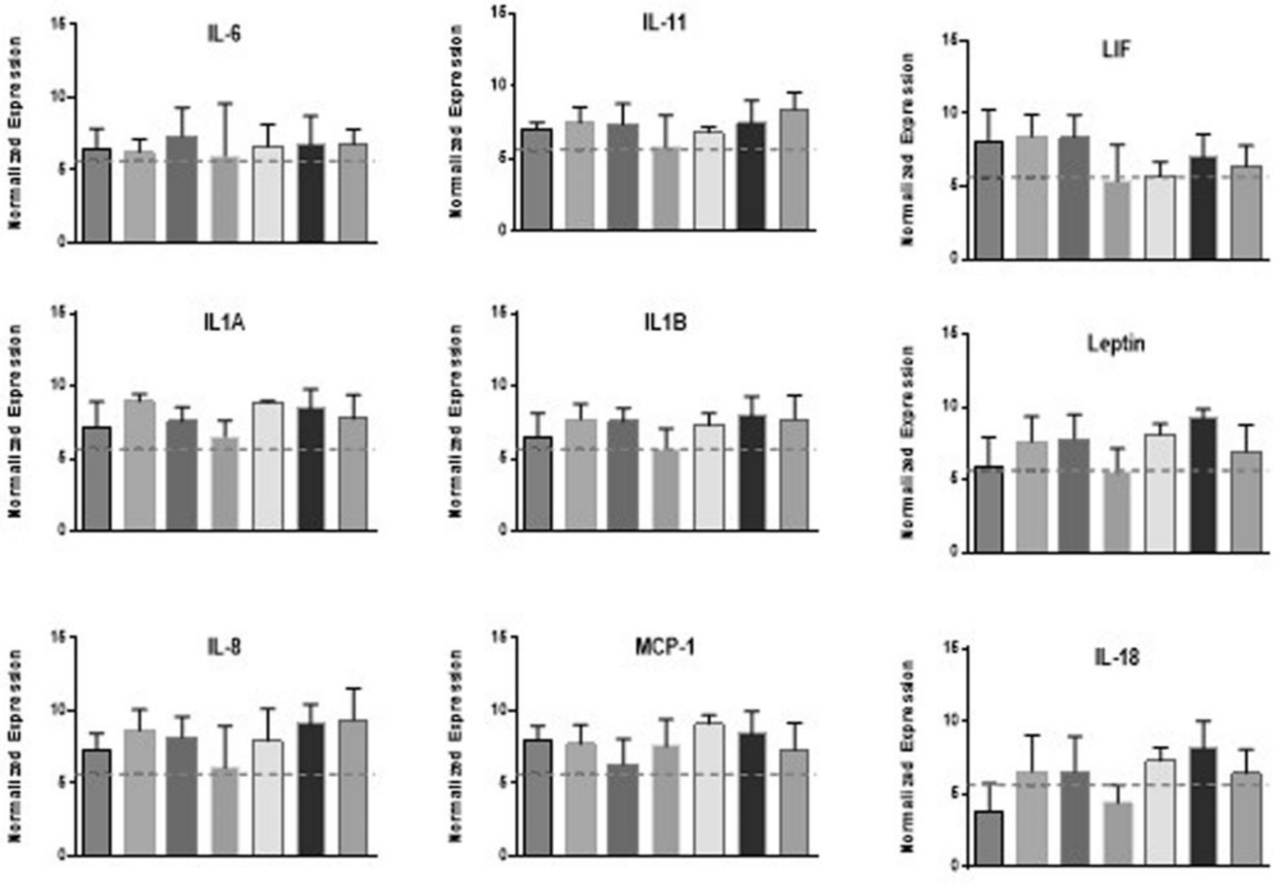

Supplementary Figure S3 Expression of cytokines in human preimplantation embryos. Microarray data (Smith et al., 2019) showing the relative expression of cytokines in individual preimplantation human embryos from oocyte to blastocyst (n = 3 at 8 cell, n = 4 for all other stages), in isolated 8 cell stage blastomeres (blastomeres n = 8) and in TE) and ICM samples isolated from blastocysts (n = 6 paired samples). Microarray data were normalised with MAS 5 and the threshold level for gene expression above background was set at 5.64 (dashed horizontal line); values <5.64 are considered as no expression, values >5.64 are positive gene expression. Data are presented as the mean ± SEM.

Human Reproduction, pp. 1–1, 2021
doi:10.1093/humrep/deab188

human reproduction

SUPPLEMENTARY DATA

Supplementary Table SI Primers used for polyAPCR in human preimplantation embryos.

Gene names	Gene abbreviation	Accession numbers	Forward 5′–3′	Reverse 5′–3′
Toll-like receptor 1	*TLR1*	NM_003263.3	AGCTGACAGAGCAAGCAAAGA	GAACTGCGACCCGAAGGTAT
Toll-like receptor 2	*TLR2*	XM_005263197	TGGCCACAAAAGGCATTCTC	TGGGGAGTGCCCCAAATACT
Toll-like receptor 3	*TLR3*	NM_003265	ACCATGCACTCTGTTTGCGA	GGCCAGTTCAAGATGCAGTG
Toll-like receptor 4	*TLR4*	NM_003266	CCCTGGGTGTGTTTCCATGT	TGCGGACACACACACTTTCA
Toll-like receptor 5	*TLR5*	NM_003268	GCCCTTGCTGGACCTACATT	AGAAGCCCTGACATCCTTGG
Toll-like receptor 6	*TLR6*	NM_006068	GTAGGATGCGCGCTTTTGTT	AAACGACGGCAGGGAAGATG
Toll-like receptor 7	*TLR7*	NM_016562	ACAGCGTGCATGTGTTCAAG	GCCACACGTGAGGAAAATACG
Toll-like receptor 8	*TLR8*	NM_016610	AAAACCGAACGCAACCACAG	TGCCACTGTGACTAATGGTCC
Toll-like receptor 9	*TLR9*	NM_017442	AATAGCCGTGAGCCGGAATC	CTGCTCTGTGTCAGGTGTGG
Toll-like receptor 10	*TLR10*	XM_011513761	ACATAGAAATCAAATGCTCCCTGT	AACAGTGGTTTGGGTCTGGG
Nuclear factor-kappa-B inhibitor alpha	*NFKBIA*	NM_020529	TGTGCTTCGAGTGACTGACC	TCACCCCACATCACTGAACG
Tumour necrosis factor receptor (TNFR)-associated factor 6	*TRAF6*	NM_004620	ATGCACGGAGCGCATAAAAC	TCCGAGGTTTCACTGCCATT
Monocyte chemotactic protein-1	*MCP-1*	NM_002982	GACCACCTGGACAAGCAAAC	TGTCTGGGGAAAGCTAGGGG
NLR family pyrin domain containing 1	*NLRP-1*	NM_001033053	CTGGATGCCTGCTTTTGTGG	TGGTGCATTTCTCTGCCTTCT
Hyaluronan mediated motility receptor	*HMMR*	NM_001142556	ACAGGTTCTTAGGCTCCATCC	AGAATGCAGGCTTAAAGGCCA
Hyaluronidase-1	*HYAL1*	NM_033159	ACTGCAGCAATCACAAAGGC	AAACGCTTAGCACGGGGATT
β Actin	*β Actin*	NM_001017992	AAGCCACCCCACTTCTCTCT	CTATCACCTCCCCTGTGTGG
