# Supplementary material for: The expression and activity of Toll-like receptors in the preimplantation human embryo suggest a new role for innate immunity
Source: Hum Reprod. 2021 Sep 13;36(10):2661–75. doi: 10.1093/humrep/deab188 (PMC8450873; doi:10.1093/humrep/deab188)
Supplement: deab188_Supplementary_Figure_S2 [file deab188_supplementary_figure_s2.pdf]

**Human Reproduction, pp. 1–1, 2021**
doi:10.1093/humrep/deab188

human reproduction

# SUPPLEMENTARY DATA

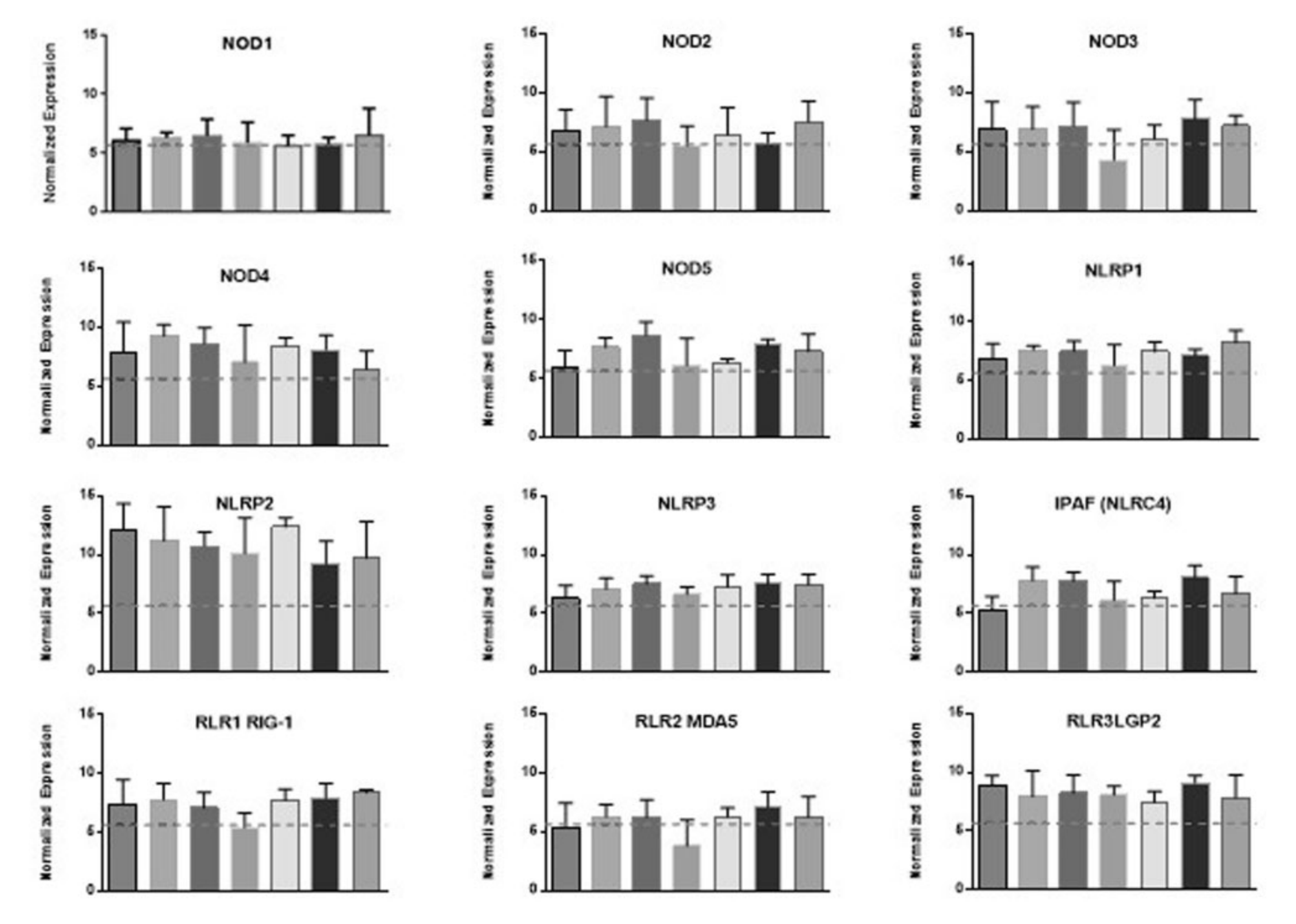


**Supplementary Figure S2** **Expression of NLR and RLR genes in human embryos.** Microarray data (Smith et al., 2019) showing the relative expression of the NLR (nucleotide-binding oligomerization domain (NOD)-like receptor) and RLR (retinoic acid-inducible gene-1, RIG-1-like receptor) molecules in individual preimplantation human embryos from oocyte to blastocyst (n=3 at 8 cell, n=4 for all other stages), in isolated 8 cell stage blastomeres (blastomeres n=8) and in trophectoderm (TE) and inner cell mass (ICM) samples isolated from blastocysts (n=6 paired samples). Microarray data were normalised with MAS 5 and the threshold level for gene expression above background was set at 5.64 (dashed horizontal line); values <5.64 are considered as no expression, values >5.64 are positive gene expression. Data are presented as the mean ± SEM.
