# Supplementary material for: The expression and activity of Toll-like receptors in the preimplantation human embryo suggest a new role for innate immunity
Source: Hum Reprod. 2021 Sep 13;36(10):2661–75. doi: 10.1093/humrep/deab188 (PMC8450873; doi:10.1093/humrep/deab188)
Supplement: deab188_Supplementary_Figure_S3 [file deab188_supplementary_figure_s3.pdf]

Human Reproduction, pp. 1–1, 2021
doi:10.1093/humrep/deab188

human
reproduction

SUPPLEMENTARY DATA

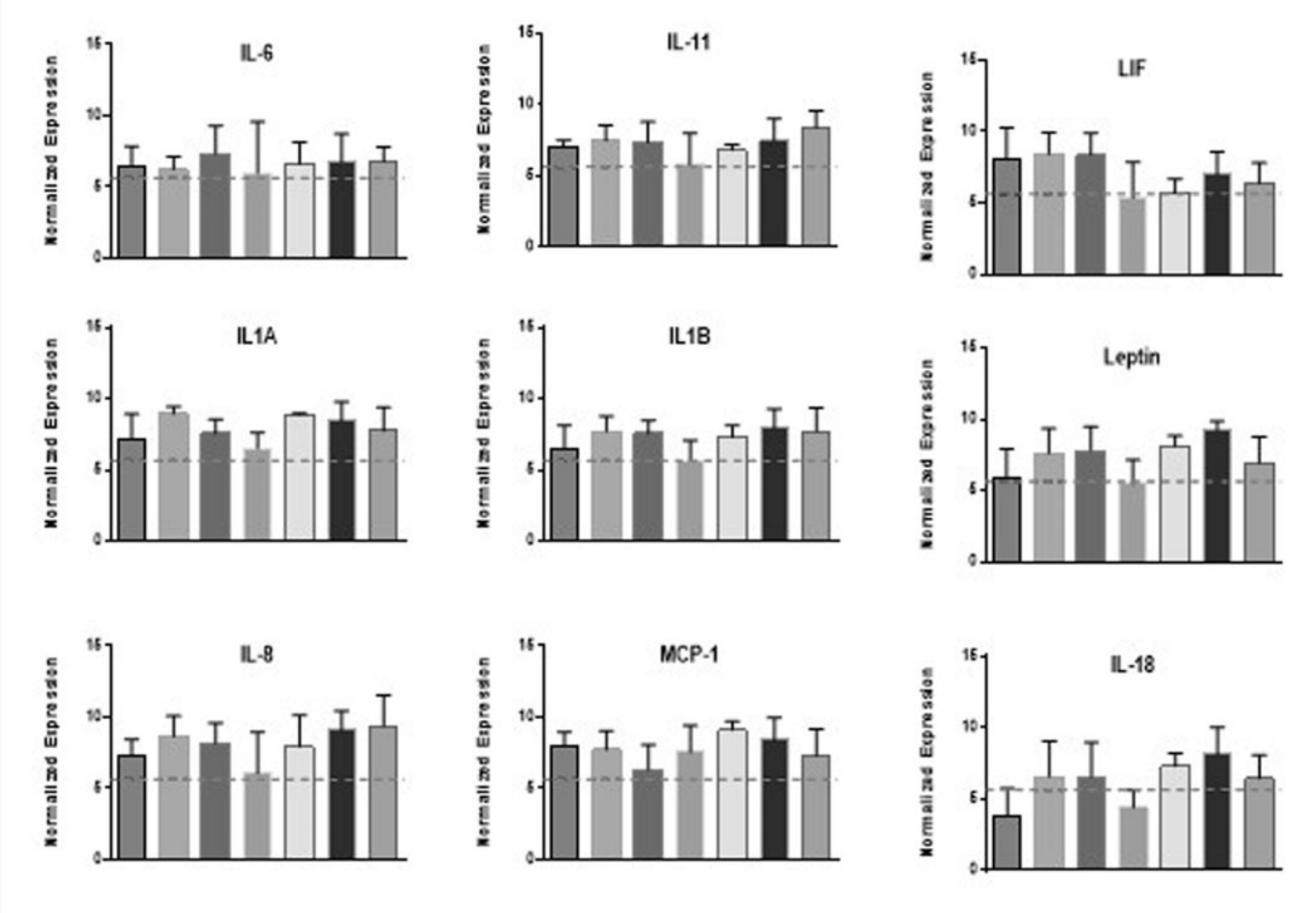

Supplementary Figure S3 **Expression of cytokines in human preimplantation embryos.** Microarray data (Smith et al., 2019) showing the relative expression of cytokines in individual preimplantation human embryos from oocyte to blastocyst (n = 3 at 8 cell, n = 4 for all other stages), in isolated 8 cell stage blastomeres (blastomeres n = 8) and in TE) and ICM samples isolated from blastocysts (n = 6 paired samples). Microarray data were normalised with MAS 5 and the threshold level for gene expression above background was set at 5.64 (dashed horizontal line); values <5.64 are considered as no expression, values >5.64 are positive gene expression. Data are presented as the mean ± SEM.
