# Supplementary material for: The expression and activity of Toll-like receptors in the preimplantation human embryo suggest a new role for innate immunity
Source: Hum Reprod. 2021 Sep 13;36(10):2661–75. doi: 10.1093/humrep/deab188 (PMC8450873; doi:10.1093/humrep/deab188)
Supplement: deab188_Supplementary_Table_S1 [file deab188_supplementary_table_s1.pdf]

Human Reproduction, pp. 1–1, 2021
doi:10.1093/humrep/deab188

human reproduction

SUPPLEMENTARY DATA

Supplementary Table SI **Primers used for polyAPCR in human preimplantation embryos.**

Gene names	Gene abbreviation	Accession numbers	Forward 5′–3′	Reverse 5′–3′
Toll-like receptor 1	*TLR1*	NM_003263.3	AGCTGACAGAGCAAGCAAAGA	GAACTGCGACCCGAAGGTAT
Toll-like receptor 2	*TLR2*	XM_005263197	TGGCCACAAAAGGCATTCTC	TGGGGAGTGCCCCAAATACT
Toll-like receptor 3	*TLR3*	NM_003265	ACCATGCACTCTGTTTGCGA	GGCCAGTTCAAGATGCAGTG
Toll-like receptor 4	*TLR4*	NM_003266	CCCTGGGTGTGTTTCCATGT	TGCGGACACACACACTTTCA
Toll-like receptor 5	*TLR5*	NM_003268	GCCCTTGCTGGACCTACATT	AGAAGCCCTGACATCCTTGG
Toll-like receptor 6	*TLR6*	NM_006068	GTAGGATGCGCGCTTTTGTT	AAACGACGGCAGGGAAGATG
Toll-like receptor 7	*TLR7*	NM_016562	ACAGCGTGCATGTGTTCAAG	GCCACACGTGAGGAAAATACG
Toll-like receptor 8	*TLR8*	NM_016610	AAAACCGAACGCAACCACAG	TGCCACTGTGACTAATGGTCC
Toll-like receptor 9	*TLR9*	NM_017442	AATAGCCGTGAGCCGGAATC	CTGCTCTGTGTCAGGTGTGG
Toll-like receptor 10	*TLR10*	XM_011513761	ACATAGAAATCAAATGCTCCCTGT	AACAGTGGTTTGGGTCTGGG
Nuclear factor-kappa-B inhibitor alpha	*NFKBIA*	NM_020529	TGTGCTTCGAGTGACTGACC	TCACCCCACATCACTGAACG
Tumour necrosis factor receptor (TNFR)-associated factor 6	*TRAF6*	NM_004620	ATGCACGGAGCGCATAAAAC	TCCGAGGTTTCACTGCCATT
Monocyte chemotactic protein-1	*MCP-1*	NM_002982	GACCACCTGGACAAGCAAAC	TGTCTGGGGAAAGCTAGGGG
NLR family pyrin domain containing 1	*NLRP-1*	NM_001033053	CTGGATGCCTGCTTTTGTGG	TGGTGCATTTCTCTGCCTTCT
Hyaluronan mediated motility receptor	*HMMR*	NM_001142556	ACAGGTTCTTAGGCTCCATCC	AGAATGCAGGCTTAAAGGCCA
Hyaluronidase-1	*HYAL1*	NM_033159	ACTGCAGCAATCACAAAGGC	AAACGCTTAGCACGGGGATT
β Actin	*β Actin*	NM_001017992	AAGCCACCCCACTTCTCTCT	CTATCACCTCCCCTGTGTGG
